# MAP‐AD Test robustness for blood mitochondrial methylcytosines measurement

**DOI:** 10.1002/alz70856_098536

**Published:** 2025-12-24

**Authors:** Marta Blanch, Beatriz Fontal, Pau Martí, Victor Fuentes, Carla Trapero, Jose Luis Mosquera, Marta Barrachina

**Affiliations:** ^1^ ADmit Therapeutics SL, Barcelona, Barcelona, Spain

## Abstract

**Background:**

The MAP‐AD Test is an IVD medical device composed of a reagent kit and medical software that integrates a classifier model for AD dementia (ADD) prognosis measuring mitochondrial methylcytosines in Mild Cognitive Impairment patients.

As part of the analytical validation, several experiments are presented to demonstrate the suitability of the analytical method used for its intended purpose.

**Method:**

To verify that MAP‐AD Test oligos maintain their performance characteristics after shipping, a batch of oligos was subjected to two scenarios, including temperatures ranging from ‐20°C to 40°C. Quality control and functionality testing of the oligos were performed using 3 replicates of 3 bisulfited DNA samples analyzed by PCR for the target amplicons, D‐Loop and ND1.

A sample stability study was performed to verify the stability of mitochondrial DNA (mtDNA) methylation levels in D‐Loop and ND1 after exposing blood samples from 5 patients to room temperature (RT) for 0, 2, 6 and 12 days prior to DNA extraction.

A site‐to‐site reproducibility study was performed at ADmit (Spain) and Eurofins Clinical Enterprise (USA), analyzing 8 DNA replicates from 10 patients at the two facilities. Differential methylation analysis (DMA) was used to identify differentially methylated sites associated with sample repeatability when comparing different replicates in D‐Loop and ND1.

**Result:**

MAP‐AD Test oligos stored at ‐20°C to 40°C amplified D‐Loop and ND1 under all tested conditions, ensuring product performance under defined shipping conditions.

The mtDNA methylation is consistent across patients and RT blood exposure times. The maximum blood exposure at RT is 11 days since mtDNA target sites are unaffected for at least 12 days.

The DMA including 8 replicates from 10 patients sequenced in 2 facilities shows adjusted *p*‐values in all cytosine with a FWER>0.05, demonstrating that the methylation analysis is repeatable and reproducible.

**Conclusion:**

The MAP‐AD Test is an accurate and reliable tool, as demonstrated by the results of its analytical validation. This is the first epigenetic IVD Test for the prognosis of ADD and highly contributes to approaching AD in a holistic manner towards personalized medicine global AD strategy, along with new therapies and biomarkers development.

